# Mouse Models of Sjögren’s Syndrome with Ocular Surface Disease

**DOI:** 10.3390/ijms21239112

**Published:** 2020-11-30

**Authors:** Sharmila Masli, Darlene A. Dartt

**Affiliations:** 1 Department of Ophthalmology, Boston University School of Medicine, Boston, MA 02118, USA; 2 Schepens Eye Research Institute/Massachusetts Eye and Ear, Department of Ophthalmology, Harvard Medical School, Boston, MA 02114, USA

**Keywords:** Sjögren’s syndrome, lacrimal gland, cornea, conjunctiva, inflammation

## Abstract

Sjögren’s syndrome (SS) is a systemic rheumatic disease that predominantly affects salivary and lacrimal glands resulting in oral and ocular dryness, respectively, referred to as sicca symptoms. The clinical presentation of ocular dryness includes keratoconjunctivitis sicca (KCS), resulting from the inflammatory damage to the ocular surface tissues of cornea and conjunctiva. The diagnostic evaluation of KCS is a critical component of the classification criteria used by clinicians worldwide to confirm SS diagnosis. Therapeutic management of SS requires both topical and systemic treatments. Several mouse models of SS have contributed to our current understanding of immunopathologic mechanisms underlying the disease. This information also helps develop novel therapeutic interventions. Although these models address glandular aspects of SS pathology, their impact on ocular surface tissues is addressed only in a few models such as thrombospondin (TSP)-1 deficient, C57BL/6.NOD.*Aec1Aec2*, NOD.H2^b^, NOD.Aire KO, and IL-2Rα (CD25) KO mice. While corneal and/or conjunctival damage is reported in most of these models, the characteristic SS specific autoantibodies are only reported in the TSP-1 deficient mouse model, which is also validated as a preclinical model. This review summarizes valuable insights provided by investigations on the ocular spectrum of the SS pathology in these models.

## 1. Introduction

Sjögren’s syndrome is a chronic inflammatory, autoimmune disease that predominantly affects exocrine glands like salivary and lacrimal glands. Functional loss of these glands leads to clinical manifestations of dry mouth (xerostomia) and dry eye (keratoconjunctivitis sicca). Such oral and ocular clinical manifestations occur in isolation as in primary Sjögren’s syndrome (pSS) or as part of other chronic inflammatory diseases (e.g., rheumatoid arthritis (RA), systemic lupus erythematosus (SLE), systemic sclerosis) as in secondary Sjögren’s syndrome (sSS) [1,2]. Although dryness of oral and ocular mucosa is the main symptom, a majority of primary Sjögren’s patients also experience systemic manifestations involving visceral organs as well as musculoskeletal and neurologic symptoms. Therefore non-specific manifestations like arthralgias and myalgias are common in SS patients resulting in delayed diagnosis [3]. While oral dryness results in complications like dental caries, chronic candidiasis, and swollen parotid glands, ocular dryness can lead to corneal melts, uveitis, scleritis, and optic neuritis [1,3].

Even though the etiology of SS remains obscure, the pathogenesis is believed to be multifactorial with the involvement of environmental, genetic and immunologic factors. Thus it is possible that environmental factors trigger inflammatory immune responses in genetically predisposed individuals. Inflammatory mononuclear infiltrates in salivary and lacrimal glands are characteristic histopathologic findings in SS patients. Due to the technically challenging nature of lacrimal gland biopsy in humans, clinicians rely on minor salivary gland (MSG) biopsy for SS diagnosis. Consequently, only a handful of studies published since 1970 include lacrimal gland histology from SS patients [3].

Lacrimal gland dysfunction in SS compromises the aqueous component of the tear film, which contributes to ocular dryness. The secretory function of the lacrimal gland is dependent on neuronal stimuli initiated at the ocular surface through afferent nerves of the cornea and conjunctiva that are relayed to the central nervous system and delivered to the lacrimal gland through efferent nerves. Thus ocular surface tissues are an integral component of the tear-secreting “lacrimal functional unit,” and functional impairment of ocular surface nerves also reduces tear secretion [4]. In pSS patients, such impaired corneal nerves were detected by in vivo confocal microscopy [5]. In addition to lacrimal glands, ocular surface tissues such as the cornea and conjunctiva are also critical for regulating tear volume. Epithelial cells of both cornea and conjunctiva can transport electrolytes and fluid and thus contribute to the aqueous component of tears [6]. Conjunctival goblet cells also secrete the soluble gel-forming mucin MUC5AC that is important for the hydration and lubrication of the ocular surface [7]. Activation of afferent corneal nerves stimulates MUC5AC secretion from goblet cells using similar efferent neural pathways as used by the lacrimal gland [8].

Ocular dryness and related symptoms of burning sensation, grittiness, and redness also result from the damaged ocular surface tissues [1]. Their functional impairment renders the ocular surface vulnerable to environmental factors and resulting inflammation further contributes to the damage and related symptoms. In the early stages of SS, these symptoms are mild and cause transient episodes of blurred vision with exacerbation by environmental factors such as heat, low humidity, or wind. The severity of symptoms progressively increases, causing severe pain, ocular surface damage, and chronic visual impairment that significantly reduce the quality of life of SS patients [1,9]. The ocular dryness results in the development of small superficial erosions of the corneal epithelium (superficial keratitis) and inflammation of the conjunctiva (conjunctivitis). Reduced tears and a damaged corneal barrier in SS patients increase the risk of bacterial and fungal infections. With increasing severity of inflammation, sterile or non-infectious corneal ulcers may also form, leading to complications including corneal melts and perforation. In moderate to severe forms of ocular dryness, thick rope-like strands of mucus are formed that trap debris from shed epithelial cells and adhere to damaged areas of the corneal epithelium (filamentary keratitis). With each blink, these mucous filaments cause severe pain and foreign body sensation. In severe cases of pSS, keratinization of both cornea and conjunctiva develops [1,3,9]. Overall changes to ocular surface tissue integrity in SS patients are closely linked to the secretory dysfunction of the lacrimal gland and, to some extent, the conjunctival and corneal epithelia.

Diagnosis of SS is made based on both oral and ocular symptoms and clinical signs. Several classification criteria have been designed to help guide clinical diagnoses of SS that included both subjective tests of symptoms and objective tests of clinical signs for both oral and ocular manifestations. The American-European consensus group criteria developed in 2002, as well as that developed by the International Sjögren’s Syndrome Criteria Working Group as American College of Rheumatology (ACR)/European League Against Rheumatism (EULAR), published in 2016, include objective tests like salivary gland biopsy, salivary flow rate, anti-SSA, and anti-SSB antibodies, ocular surface assessment and tear secretion [10]. Objective tests that evaluate the ocular surface integrity involve grading the ocular staining score using vital dyes like Rose-bengal or Lissamine green that detect damaged conjunctival epithelium or Fluorescein that detects damaged corneal epithelium. Tear secretion is assessed by the Schirmer test for quantitative tear measurement. This is achieved by placing a test strip of filter paper near the lower conjunctival sac to determine the length of the paper wetted by tears after 5 min. Schirmer I is performed without local anesthesia to measure baseline and reflex tears, whereas Schirmer II is performed with anesthetic to measure baseline tears. Subjective tests for ocular dryness include questionnaires probing dry eye symptoms and quality of life. Several questionnaires have been developed and validated [3]. Patient responses to these questions help distinguish those with dry eyes from normal individuals, while also determining the severity of symptoms and their impact on the vision.

In research studies significantly reduced conjunctival goblet cells are detected in impression cytology samples from SS patients indicating damage to the ocular surface [11,12,13]. To achieve non-invasive enumeration of goblet cells, in vivo confocal microscopy is employed [14,15]. Both of these methods are not yet standardized. Significant variations reported in goblet cell numbers exist between studies due to variations in protocols and sampling locations on the conjunctiva.

Measurement of tear composition and tear function is considered valuable in diagnosing ocular dryness [16]. Changes in tear composition are reflected in the tear film’s elevated osmolarity, while abnormal tear function resulting from ocular surface irregularities is detected in a tear stability indicator test and tear breakup time (TBUT). The latter is proposed to differentiate non-SS pathology underlying ocular dryness [17]. A recent study estimated the sensitivity and specificity of commonly used clinical signs and tests in the diagnosis of dry eye, keratoconjunctivitis sicca (KCS), associated with SS. This study identified the ocular staining score to be highly sensitive and specific in distinguishing populations with KCS from those with non-SS dry eye [18]. Therefore, evaluation of the damage to corneal and conjunctival epithelia represent a highly useful objective measures in diagnosing SS related ocular dryness and assessing the integrity of the lacrimal functional unit.

## 2. Mouse Models

Animal models provide valuable tools that can mimic complexities of the human body that are difficult to achieve in any in vitro model and therefore remain irreplaceable in any study of human disease. These models not only facilitate a closer understanding of pathogenic mechanisms of human disease but also help explore the efficacy of candidate drugs and predict responses in patients. The mouse is the most common species used in human disease research due to its striking genetic homologies with humans, rapid breeding, and availability of inbred strains [19]. Also, emerging advanced approaches to manipulate the mouse genome have provided powerful tools to develop new mouse models. No mouse model, however, can replicate the full spectrum of phenotypes in a given human disease. Likewise, it is not possible to recapitulate every aspect of a complex disease like SS in a single mouse model. The majority of SS mouse models were developed to recapitulate salivary (SG) and lacrimal gland (LG) pathology and related loss of saliva and tear secretion, respectively. These models have been extensively reviewed [20,21,22]. In very few models, ocular surface tissues that are an integral part of the lacrimal functional unit with a direct impact on tear quantity and quality have been investigated and characterized. Herein, we will review SS models in which the development of ocular surface disease has been characterized (Table 1). We have not included the desiccating stress mouse model, as it is not a model of SS but rather of dry eye. As one of the uses of mouse models is in clinical translation, similarities to human disease require validation in proof-of-principle experiments demonstrating a response to treatment [23]. We will also describe the utility of SS models for use as preclinical models to advance translational research in SS and KCS, the ocular component of SS.

### 2.1. Thrombospondin-1 (TSP-1) Deficient Mice

The matricellular glycoprotein, TSP-1, is expressed by several cell types, including those in ocular tissues and the LG [24,25]. It is the first identified activator of latent TGFβ to function in vivo and there are similarities in TSP-1 and TGFβ1 deficient mice. The ability of TSP-1 deficient mice to avoid the early post-natal death observed in TGFβ1 deficient mice clearly points to the existence of additional TGFβ activation pathways that do not rely on TSP-1 [26]. Inflammation in multiple organs reported in TSP-1 deficient (TSP-1−/−) mice is consistently at a lower level as compared to that seen in TGFβ1 deficient mice. The ability of TSP-1 to activate latent TGFβ2 (that cannot be activated by integrins) and the predominant expression of TGFβ2 in ocular tissues and LG (unpublished observations) correlates with the spontaneous development of ocular manifestations in TSP-1−/− mice [24]. Moreover, TSP-1 additionally functions to inhibit heme- and lymphangiogenesis and plays a critical role in wound healing. For a review of these additional functions, see Masli et al. (2014) [24]. The multiple and diverse functions of TSP-1 highlight its significance in the homeostasis of ocular surface tissues and ocular adnexa.

Mice deficient in TSP-1 develop primary SS characterized by mononuclear infiltrates of LG [27] and SG (unpublished data) as well as SS related autoantibodies (anti-SSA and anti-SSB). Lacrimal gland pathology in these mice is characterized extensively in the context of ocular surface manifestations that include ocular surface dryness resulting in KCS. Due to the insidious nature of SS in humans, mild early symptoms are easily overlooked and misinterpreted, causing a delay of several years in confirmed SS diagnosis. Therefore mean age at the time of SS diagnosis is over 50 years, with an onset usually reported in the fourth to fifth decade [28]. Temporal analysis of clinical and pathological manifestations in TSP-1−/− mice identified a similar insidious pattern with an established disease detectable in 12–14-week-old mice, which corresponds to the fourth to fifth decade in human age [29]. Thus, the development of SS in TSP-1−/− mice closely recapitulates human disease.

Accumulating evidence suggests that the expression of TSP-1 is also relevant in SS related pathology in patients. In one study, significantly reduced expression of TSP-1 in SG of SS patients was correlated with severe inflammatory infiltrates detected in these glands [30]. Similarly, reduced TSP-1 expression in ocular surface epithelial cells and related genetic polymorphism in the TSP-1 encoding gene was associated with a significantly increased risk of developing chronic ocular surface inflammation [31]. The relevance of TSP-1 in corneal inflammation was highlighted in another study that reported a significant correlation of TSP-1 polymorphism with the increased risk of corneal transplant rejection [32]. Another recent study also reported decreased serum TSP-1 in SLE, a disease frequently reported in SS patients [33]. These human studies suggest that TSP-1−/− mice can help unravel potential mechanisms underlying SS pathogenesis that is likely to be common in both primary and secondary disease in humans.

In TSP-1−/− mice, early ocular surface and adnexal clinical signs are detectable in 8–12-week-old mice prior to the development of typically diagnostic glandular mononuclear infiltrates and SS specific autoantibodies [27]. At present, this is the only SS model with an ocular surface disease that is validated based on proof-of-principle experiments demonstrating therapeutic response to topically administered steroids, which represents the current standard of care [34]. This study, along with the relevance of TSP-1 to SS in humans, strongly supports the utility of TSP-1−/− mice as a suitable preclinical model to evaluate the therapeutic efficacy of novel treatments.

Both male and female TSP-1−/− mice develop SS related pathology. However, incidents of severe ocular surface changes are seen more frequently in female mice, suggesting increased intensity of the disease in female mice. In some TSP-1 deficient mice, ocular surface disease becomes progressively severe with corneal histology that resembles corneal melt with sterile inflammation (unpublished observations). Such pathology is also reported in humans with severe cases of SS. In some TSP-1−/− mice, the severe ocular surface disease is followed by the loss of the eyeballs (phthisis) [27]. The increased severity of the ocular surface disease noted in female TSP-1−/− mice are further supported by LG histology studies, in which inflammatory infiltrates in female mice were detected at a younger age (12 weeks) as compared to those reported in male mice (24 weeks) [35]. These findings are consistent with the widely reported overall sex bias in autoimmunity, as well as the observed higher frequency of oral and ocular dryness in female SS patients [36]. The gender differences in clinical manifestations noted in TSP-1 deficient mice also point to a potential role of estrogen-dependent mechanisms underlying SS pathology in this model that remain to be addressed [37]. Further details covering the analysis of SS pathogenesis in these mice are described below.

#### 2.1.1. Lacrimal Gland Pathology

Several aspects of LG pathology that contribute to their functional loss are extensively characterized in TSP-1−/− mice. These include degenerative changes and inflammatory infiltrates, abnormalities of repair and regeneration, and the disruption of neural regulation of the lacrimal functional unit.

##### Loss of Secretory Function

Functional loss of LG is detected in TSP-1−/− mice based on their significantly reduced ability to secrete protein (peroxidase) in response to neural stimulation both ex vivo and in vivo, although tear volume remains unaltered. Such compromised protein secretory function of LG correlates with the structural damage to secretory units and apoptosis of epithelial cells detected in the gland [27]. The volume of tear secretion reflects both secretions from the LG and ocular surface epithelial cells as well as leakage from the conjunctival blood vessels. It is possible that the unaltered tear volume in TSP-1−/− mice represents inflammation and fluid leakage from conjunctival blood vessels that obscure changes in epithelial fluid secretion.

##### Degenerative Changes and Inflammatory Infiltrates

Progression of LG pathology occurs earlier in female TSP-1−/− mice than in male mice. This is indicated by inflammatory cytokine expression and inflammatory cell infiltrates [35]. Lymphocytic infiltrates in LGs of female mice are detected at as early as 12 weeks of age in contrast to male mice that do not exhibit LG infiltrates until 24 weeks of age [27,35]. However, degenerative changes in LGs of both male and female TSP-1−/− mice are detected prior to the appearance of inflammatory infiltrates. In 8-week-old male mice, an increased proportion of LG epithelial cells undergo apoptosis [27]. This observation prompted an evaluation of degenerative changes that occur with disease progression [35]. Secretory units of LG, acini, in 12 week-old female TSP-1−/− mice are significantly reduced in size compared to those in wild-type (WT) LGs. Their subcellular structure is altered, in that acini in TSP-1−/− LGs contain more vacuoles, and the amount of lysosomes, mitochondria, and secretory granules, but not endoplasmic reticulum, is decreased. Concomitant with these changes is the decline in protein secretion response of LGs as determined in vitro. Thus in TSP-1−/− mice, both degenerative changes and the related loss of secretory function of the LG precede the appearance of inflammatory infiltrates. This finding suggests that the systemic immune response in TSP-1−/− mice likely develops against antigenic material released from degenerating cells. Also, long before the infiltration of glands, the expression of inflammatory cytokines IL-1β, IL-6, and IFN-γ is detected in LGs of younger TSP-1−/− mice [27,35]. Presumably, resident immune cells of the gland are the source of these early inflammatory cytokines that are also likely to contribute to the observed structural deterioration. In addition to degenerative changes, cellular proliferation and the expression of CD47 receptor that permits integrin-mediated proliferation is significantly reduced in LGs of female TSP-1−/− mice. Furthermore, the expression of additional genes associated with proliferation is decreased, including the pluripotency factor Oct4 and the regulators of cell proliferation and maintenance including Tert, Runx1, Pax6, Klf4, Klf5, Notch 2, Sox6, and Sox 9 [35]. Collectively degenerative changes accompanied by the decreased proliferative capacity of LG cells underlies the disease progression in TSP-1−/− mice.

##### Abnormalities of Glandular Repair and Regeneration

**Figure 1:**
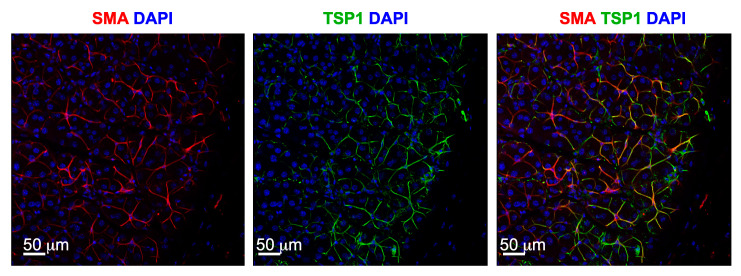
Myoepithelial cells in LGs express TSP-1 Fluorescence microscopic images were captured from mouse LG section immunostained for MEC marker—smooth muscle actin (SMA) in red, TSP-1 (green) and nuclear stain DAPI (blue). Images were kindly provided by Dr. Helen P. Makarenkova, Scripps Research, La Jolla, CA, USA.

Further supporting the LG degeneration in TSP-1−/− mice is a change in progenitor cells. Immunostaining of several stem cell markers including ABCG2, Nestin, Musashi 1, Sox2, Pax 6, CHX10, and deltaNP63 identify the myoepithelial cells (MECS) as potential progenitor cells in LG [35]. These cells surround the acinar cells and contract upon stimulation. Their contractile role will be discussed later in this review. After glandular damage, MECs are demonstrated to contribute to LG repair by proliferating to form acinar cells [38]. Interestingly, TSP-1 is localized to the MECs, suggesting a major role for TSP-1 in their function (Figure 1). Evaluation of the amount and location of progenitor cell markers in LGs of female TSP-1−/− mice compared to WT mice shows an increased expression of ABCG2 and nestin, but a decreased expression in Musashi-1 and Sox 2. These changes likely impact the LG MEC regenerative capabilities disrupting repair of glandular damage in TSP-1−/− mice, thereby contributing to the disease progression. This possibility is currently being investigated.

##### Disrupted Neural Regulation of Lacrimal Functional Unit

**Figure 2:**
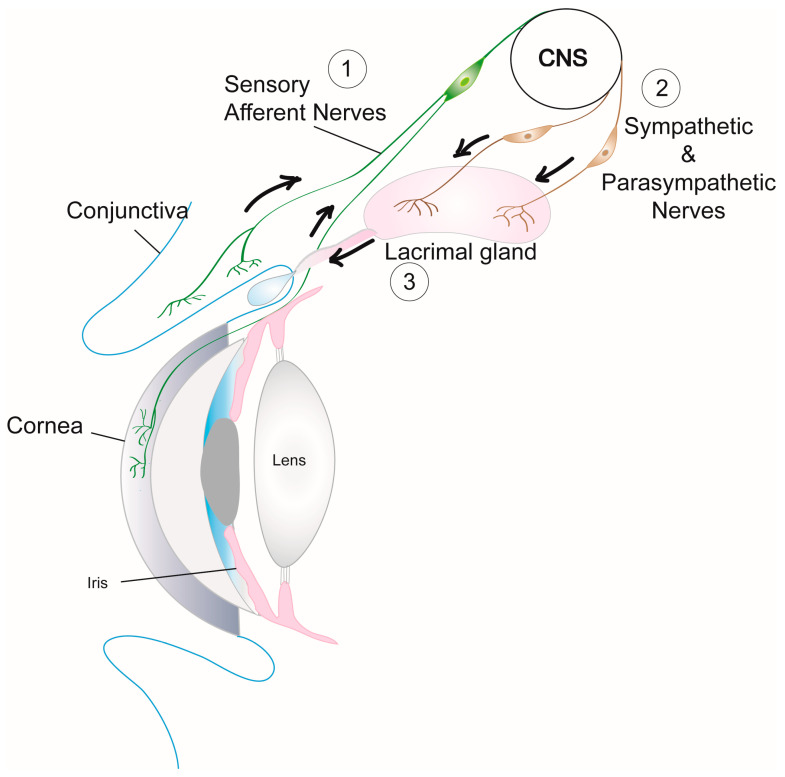
Neural regulation of the lacrimal functional unit. Schematic of the components of lacrimal Figure 1. impulses transmitted from the cornea and conjunctiva via sensory afferent nerves are received in the central nervous system (CNS) to stimulate (2) efferent sympathetic and parasympathetic nerves that innervate LG cellular components (acinar and duct epithelial cells and MECs) resulting in (3) secretion of tear fluid containing proteins, electrolytes, and water that is delivered onto the ocular surface through the duct system.

Neural regulation of the lacrimal functional unit is critical for the functional integrity of ocular surface tissues. Activation of sensory nerves in the cornea and conjunctiva by irritants in the external environment transmits impulses via the trigeminal ganglion to the central nervous system and back through the parasympathetic and sympathetic efferent nerves that innervate the LG (Figure 2) [39,40]. Neurotransmitters released from both these efferent nerve types interact with their receptors on MEC, acinar, and duct cells to increase intracellular [Ca^2+^] via several signaling pathways and cause protein as well as electrolyte and water secretion into the ocular surface tear film [4]. The disruption of the neural regulation of the lacrimal functional unit in TSP-1−/− mice contributes to the functional loss of LG and the disease progression.

(a) Abnormalities of corneal nerves: neural abnormalities are reported in the corneas of patients with SS and neuropathic corneal mechanical hypersensitivity in SS appears to be induced by ocular surface inflammation [41,42]. Although corneal sensory nerve density is lower in TSP-1−/− mice compared to WT mice, there is no change in the nerve density corresponding with the expression of inflammatory factors in the tissue [43]. Altered morphology of corneal nerves in TSP-1−/− mice, however, is concomitant with an increased expression of the pro-inflammatory cytokines MCP-1, TNFα, and MIP2. This finding is similar to observations made in SS patients. The corneal sensory nerves contain two major types of neurotransmitters CGRP and Substance P [44]. While CGRP is reported to mediate an anti-inflammatory pathway [45,46], Substance P is known to induce and mediate inflammation [47]. In TSP-1−/− mice, a significant decline in corneal nerves containing CGRP, but not Substance P, is consistent with the presence of inflammation [43]. These observations strongly implicate the contribution of inflammation-mediated impairment of corneal nerves to the loss of LG secretory function noted in TSP-1−/− mice.

(b) Abnormalities of neural response in LG: signals initiated at the ocular surface by sensory nerves are delivered to the LG via efferent nerves. In the mouse LG, the parasympathetic nerves predominate with far fewer sympathetic, and scarce sensory nerves [48]. Parasympathetic nerves surround the MECs and the basal aspect of the acini and duct cells. These cells are targeted by neurotransmitters released from the nerves to induce LG secretion. In mammary and salivary glands, contractile responses of MECs are known to stimulate secretion [49,50] and therefore MEC contractile responses in LG are likely to be critical for tear secretion. Similar to corneal nerves, the density of the overall, as well as the parasympathetic, innervation in LGs of TSP-1−/− mice is significantly reduced. This change in LGs is determined by immunostaining of a general neural marker, TUJ-1, and parasympathetic neurotransmitter, VIP, which indicates parasympathetic nerves. The decreased innervation in LGs of TSP-1−/− mice is noted regardless of the inflammation [51]. In contrast, the response of LG acini to parasympathomimetic cholinergic stimulation to secrete protein is decreased when acini are derived from TSP-1−/− LGs with inflammation. Similarly, contractile responses of MECs to stimulation with cholinergic agonist and VIP are decreased when MECs are derived from TSP-1−/− LGs with inflammation [52]. These findings implicate dysfunction of both the MECs and acini in their responses to signals received via parasympathetic nerves in causing LG functional loss in TSP-1−/− mice. Interestingly, disrupted neural regulation in LGs correlates with the presence of inflammation similar to the changes in corneal sensory nerves. Thus inflammation-driven neurologic changes appear to contribute to the impaired lacrimal functional unit and ocular surface disease progression in TSP-1−/− mice.

In summary, SS related pathological changes in LGs of TSP-1−/− mice include structural, functional, and neurological damage that impact the secretory function of LG and cause ocular surface disease. While structural abnormalities likely alter initial secretory function in younger mice (6–8 week-old), these do not cause overt ocular surface disease with inflammatory changes. Instead, these earlier structural changes in the LG correlate with the subclinical development of inflammation detected in the form of increased expression of inflammatory cytokines. Subsequently, progressive chronic inflammation leads to the development of inflammatory infiltrates and neurological damage that exacerbates LG functional loss resulting in established ocular surface disease in 12-week-old TSP-1−/− mice.

#### 2.1.2. Corneal and Conjunctival Inflammation

**Figure 3:**
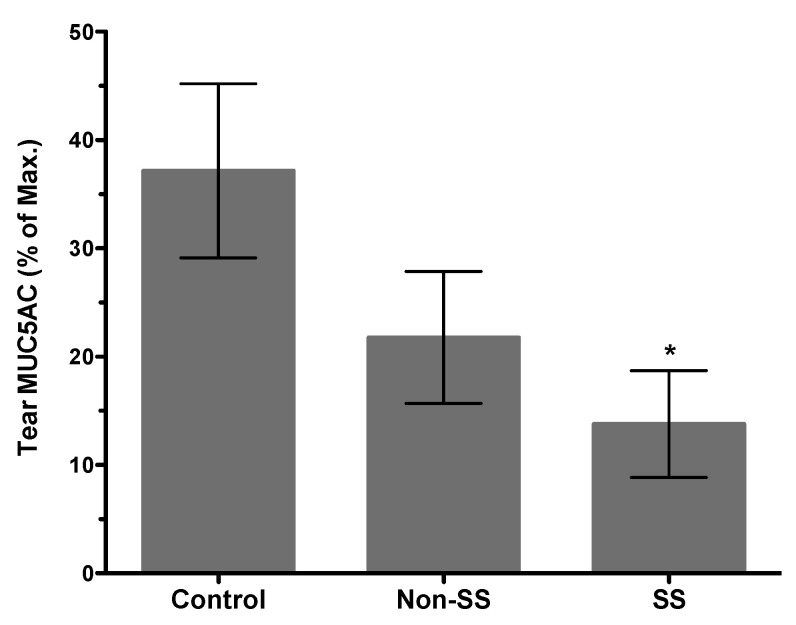
Distinctly reduced tear MUC5AC levels in patients with Sjögren’s associated dry eye. Comparison of tear MUC5AC levels in patients with non-Sjögren’s (non-SS) and Sjögren’s (SS) associated dry eye with healthy control subjects. (*n* = 20 per group, * *p* < 0.05 compared to control group).

**Figure 4:**
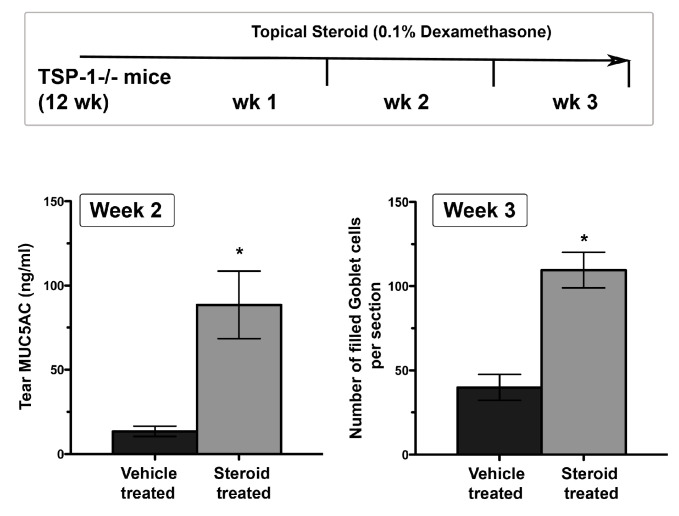
Tear MUC5AC level is a potentially sensitive indicator of anti-inflammatory efficacy of steroids relative to goblet cell density. In experiments that involved topical treatment of 12-week-old TSP-1−/− mice with 0.1% dexamethasone or vehicle (once per day) for a period of three weeks, tear MUC5AC levels recovered earlier than goblet cell density. (*n* = 5 per group, * *p* < 0.05).

Structural damage detected in LGs of young TSP-1−/− mice coincides with the subclinical inflammatory changes in the gland represented by increased expression of inflammatory cytokines IFNγ and IL-6 [27]. Similar inflammatory changes also develop in the conjunctiva, and similar to LGs they develop prior to the appearance of inflammatory infiltrates [53]. Inflammatory cytokines like IFNγ and TNFα in the conjunctiva have the ability to inhibit soluble mucin, MUC5AC, secretion by goblet cells [54]. Consistent with the inhibitory effect of these cytokines, tear MUC5AC levels are significantly reduced in TSP-1−/− mice [53]. Thus, reduced secretion of antimicrobial peroxidase, as well as soluble gel-forming mucins, together compromise the protective function of the tear film. These changes in tear quality (rather than the quantity) are also reflected in an altered commensal microbial population at the ocular surface, which may serve as an early indicator of ensuing inflammation [55]. These tear abnormalities correlate with the epithelial damage detected in TSP-1−/− mice by 12 weeks of age. The loss of corneal barrier integrity is indicated by the corneal fluorescein staining score that peaks in 12-14-week-old TSP-1−/− mice and is also accompanied by the loss of goblet cells [27,34]. Thus reduced tear MUC5AC levels in TSP-1−/− mice correlate strongly with the damage to the conjunctival and corneal epithelia. Similarly, significantly reduced tear MUC5AC levels are also detected in SS patients with dry eye (Figure 3) and are correlated with an increase in Lissamine green staining score indicative of conjunctival epithelial damage [56]. Together these results support the potential use of tear MUC5AC level as an objective measure to evaluate conjunctival goblet cell function rather than reliance on goblet cell number that requires invasive sampling of cells with impression cytology. Furthermore, in response to topical steroid treatment, goblet cell number in TSP-1−/− mice is restored after 3 weeks [34], while significant improvement in tear MUC5AC level is detected within 2 weeks of treatment (Figure 4). These findings suggest that tear MUC5AC measurement may also prove to be a relevant and valuable endpoint measurement in clinical trials to evaluate the therapeutic efficacy of novel drug candidates.

#### 2.1.3. Systemic Immune Response and Autoantibodies

In addition to glandular infiltration by Th1/Th17 cells, several studies have reported peripheral imbalance among inflammatory Th1, Th17, and regulatory Foxp3+ Treg cells in SS patients [57,58,59,60]. Likewise, evaluation of systemic immune response in TSP-1−/− mice at different stages of the disease reveals significantly increased Th1, Th17, and reduced Foxp3+ Tregs in younger mice (8–12-week-old). This change in the systemic immune response was followed by the appearance of Th17 cells in LGs of older mice (>21 weeks) [27,53]. A similar significant expansion of splenic marginal zone B cells was observed at 12 weeks, prior to the detection of anti-SSA and anti-SSB antibodies in 12–16-week-old mice [61]. Whether the increased frequency of marginal zone B cells in the LG and spleen of TSP-1−/− mice is related to lymphoma development as seen in SS patients remains to be determined.

**Figure 5:**
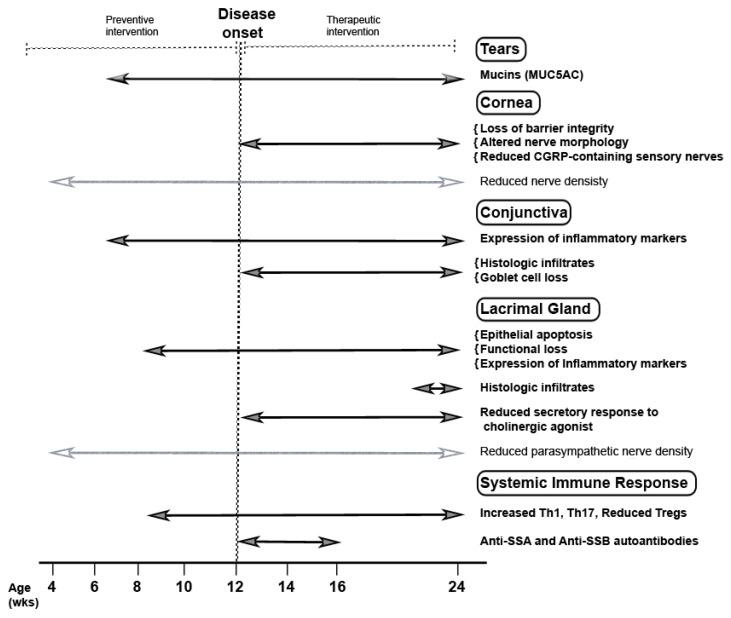
Pathologic changes that elucidate chronological and progressive development of SS related ocular surface disease in TSP-1−/− mice. Earliest changes related to SS development in Table 1. Mice are subclinical and noted at the age of 6 weeks in the form of expression of inflammatory effectors in the conjunctiva and consequential loss of tear MUC5AC content. At the age of 8 weeks, changes in the LG epithelial cells, their functional loss, and peripheral immune response are detected. These changes are followed by the disruption of corneal barrier integrity, altered corneal nerve morphology, conjunctival infiltrates, and loss of conjunctival goblet cells 12 weeks onwards and are also accompanied by the development of SS related autoantibodies. All these changes precede the appearance of histologically detectable inflammatory infiltrates in the LG. Reduced nerve densities in the cornea and LG are detected at all tested ages and appear to be independent of inflammation (identified by light gray lines with empty arrowheads). However, inflammation disrupts secretory responses of LG cells to neural stimuli and impairs lacrimal functional unit resulting in ocular surface disease. Overall, progressive changes in this preclinical model show the clear onset of ocular manifestations at 12 weeks marking two clear phases before and after the onset that allows for testing of preventive as well as therapeutic interventions, respectively.

All the investigations in TSP-1−/− mice demonstrate that they closely recapitulate SS related ocular surface disease in humans resulting from disruption of the lacrimal functional unit and systemic inflammatory responses, including SS specific autoantibodies. In this mouse model, progressive development of the ocular surface disease is characterized based on clinical signs used to evaluate ocular dryness in SS patients. The timeline of pathological changes in TSP-1−/− mice presented in Figure 5 summarizes key changes. The temporal analysis of clinical signs clearly identifies phases prior to and after the onset of ocular surface disease. Knowledge of these phases in this SS mouse model makes it a valuable tool in translational studies to evaluate both preventive and therapeutic approaches. The demonstrated efficacy of steroids in this model also validates it as a suitable preclinical mouse model of SS related ocular surface disease [34].

### 2.2. Non-Obese Diabetic (NOD) and Related Mouse Models

The NOD strain was originally developed in Japan during attempts to derive a cataract-prone strain from the outbred Jcl:ICR line. In one of the sublines, a diabetic female mouse was identified, which served as a founder for subsequent breeding. Further inbreeding of diabetogenic sublines led to the development of the NOD mouse [62]. Spontaneous development of insulitis in NOD mice is followed by the development of insulin-dependent diabetes. Based on the common manifestation of xerostomia (dry mouth) observed in diabetic patients and Sjögren’s patients, salivary glands of NOD mice were examined, followed by a functional analysis [63]. Subsequent studies demonstrated inflammatory infiltrates in the LG and reduced tear secretion and presented NOD mice as a model of SS [64]. A distinct feature of NOD mice compared to other SS models available at the time was the functional loss of both exocrine glands as determined by reduced salivary and tear secretion. The secondary SS in NOD mice has not been characterized further, possibly due to limitations imposed by the lack of availability of an appropriate non-diseased control strain for comparison. To overcome this issue and to eliminate any potential influence of diabetes on SS phenotype, subsequent studies developed several NOD strains. Of these, three strains are examined for the development of ocular surface disease.

#### 2.2.1. C57BL/6.NOD-Aec1Aec2

Genetic studies in NOD mice have identified almost 20 genetic regions or loci (*Idd* loci) that regulate genetic susceptibility in these mice to diabetes [65]. To study the effect of a single *Idd*, several *Idd* congenic strains of mice were developed, which are also helpful in identifying *Idd3* and *Idd5* as loci associated with autoimmune exocrinopathy in NOD mice. These loci were designated as Aec1 and Aec2, respectively, and incorporated in the C57BL/6 background to generate C57BL/6.NOD-Aec1Aec2 mice that do not develop diabetes [66]. Thus Aec1 (*Idd3)* and Aec2 (*Idd5)* genetic regions from NOD are sufficient for developing SS disease phenotype comparable to that detected in NOD mice and help create a primary SS model.

Original characterization of C57BL/6.NOD-Aec1Aec2 mice included LG histology reports of mononuclear infiltrates in 16-week-old mice [66]. A subsequent study evaluated LG function and ocular surface tissues [67] and reported no reduction in tear volume in 4, 12 and 20-week-old mice. In fact, tear volume is significantly increased despite the presence of infiltrates containing predominantly CD4+ T cells in LGs of 20-week-old mice, compared to younger mice. A similar age-related increase in tear volume is detected in C57BL/6 control mice. Although hyposalivation representing SG functional loss follows inflammatory infiltration of the gland [66], in LG, glandular infiltrates do not result in loss of tear volume. Consistently, there are no changes detected in the corneal permeability of C57BL/6.NOD-Aec1Aec2 mice as compared to the age-matched controls, although increased expression of IL-1β is noted in the corneas of 4-week-old mice. Increased expression of inflammatory cytokines IL-1β and TNF-α in the conjunctiva of 12-week-old C57BL/6.NOD-Aec1Aec2 mice correlates with significantly reduced goblet cell density. This study primarily focuses on evaluating age-related changes in ocular surface disease in C57BL/6.NOD-Aec1Aec2 mice and concludes that changes in the conjunctiva develop independently of LG inflammation.

#### 2.2.2. NOD.H2^b^

Several studies examining the role of MHC region in diabetes susceptibility constructed congenic strains where the MHC region of NOD mice was replaced with the corresponding region from non-diabetic strains. These studies generated a strain NOD.*H2^b^* that does not develop overt diabetes but develops glandular infiltrates that accompany reduced tear volume [68]. A study that evaluated ocular surface disease in these mice reports predominantly CD4+ T cells among LG infiltrates [69]. In this study, 4-week-old mice served as the control, and disease phenotype was assessed in 16-week-old mice. Although this comparison did not disclose a strong difference in corneal permeability, inflammation in the conjunctiva is evident based on the significantly increased CD4 and γδ TCR+ cells and reduced goblet cell density. This study focused on evaluating whether desiccating stress exacerbated ocular surface disease. Although such exacerbation was reported, ocular surface disease is also known to develop in C57BL/6 mice exposed to desiccating stress. Therefore it remains to be determined if the exacerbation in NOD.H2^b^ mice can be attributed to LG infiltrates.

#### 2.2.3. NOD.Aire Knock out

Based on the development of SS in a subset of patients with autoimmune polyglandular syndrome type 1 (APS1) that results from a defect in the *AIRE* gene, Aire-deficient mice were backcrossed into NOD mice in addition to BALB/c and C57BL/6 mice. Deficiency of Aire in the NOD background results in glandular infiltrates and severe disease compared to Aire deficiency in BALB/c background, while Aire deficient C57BL/6 mice fail to develop SS [70]. Intense infiltration of LG and cornea by predominantly CD4+ cells is detected in 6-weeks-old mice, and a consistently significant reduction in tear volume is noted in these mice compared to the Aire-sufficient NOD strain. These pathological changes and their intensities are gender-independent in this model. Changes in the cornea include detection of filamentary keratitis and increased Lissamine green staining. Such corneal changes are seen in SS patients with severe ocular surface disease. No conjunctival pathology is evaluated in this model. The density of nerves innervating corneal and LG epithelial cells is decreased in NOD.Aire KO mice [71]. This study also reported a decrease in the activity of the parasympathetic neurotransmitter, acetylcholine in LG of NOD.Aire KO mice. Wild type controls, however, are Balb/c mice. Inflammation-mediated changes in corneal innervation are demonstrated in Balb/c.Aire KO mice and not NOD.Aire KO mice. The use of NOD vs. Balb/c mice as WT controls in different studies makes it difficult to draw definitive conclusions regarding the disruption of lacrimal functional unit in this model. In another study efficacy of IL-1R antagonist is evaluated to demonstrate reversal of corneal pathology in NOD.Aire KO mice [72]. However, this model is yet to be validated with the current standard of care in human SS patients. The intense pathological changes in this model are reported to develop in young mice (6-weeks-old) with no reports on age-related progression of the disease.

### 2.3. IL-2Rα (CD25) Knock out Mice

Regulatory T cells (Tregs), CD4+CD25+, are known to play a major role in preventing autoimmunity. The deficiency of Treg cells is potentially one of the mechanisms underlying SS pathogenesis. To assess this possibility CD25KO mice were investigated for the development of SS. These mice develop SG and LG infiltrates with the loss of SG function [73]. Later LG pathology and functional loss were further characterized in the context of ocular surface tissues. While tear volume is not evaluated in these mice, LG infiltrates develop beginning at 8 weeks and increasing with age with the predominance of CD8+ cells [74,75]. Significantly increased CD4+ cells are detected in the conjunctiva beginning at 8 weeks of age. However, corneal permeability is unaltered in 8-week-old mice and is disrupted only from 12 weeks onwards. In this model, inflammatory infiltrates in either LG or conjunctiva in young mice do not appear to affect the integrity of the corneal barrier. Furthermore, decreased corneal innervation and sensitivity are observed in 4–8-week-old CD25KO mice, which are also accompanied by morphological changes in the nerves [76]. Considering the age-related disease progression reported in this mouse model, morphological changes in corneal nerves detected in young mice do not appear to be associated with inflammation as seen in SS patients. Also, the innervation and secretory response of the LG is not evaluated in this model. Therefore it remains unknown if the aberrant corneal innervation has an impact on LG responses to eventually disrupt LG functional unit.

### 2.4. Sjögren’s Syndrome Models That Do Not Address Ocular Surface Disease

There are several strains of mice that are reported to spontaneously develop exocrine gland pathology similar to that in SS patients. These include inbred strains like NOD.*H2^h4^*, NZB/NZW-F1, MRL/lpr, thymectomized NFS/Sld, and IQI/Jic [21,77,78,79,80]. In all these strains, characterization of the disease is focused on detecting mononuclear infiltrates in the SGs and LGs, demonstrating salivary hyposecretion and in some cases the presence of autoantibodies. In fact, in MRL/lpr mice the presence of infiltrates in the SG is not associated with the loss of their secretory function. In addition to inbred strains, several transgenic strains are developed that are genetically manipulated to suppress or overexpress a specific gene that regulates key molecular mechanisms and trigger SS pathology. For instance, several factors that modulate B cell responses are targeted in transgenic mice to either overexpress B cell growth factors, IL-14α or B cell-activating factor (BAFF), or fail to express Adaptor molecule Act-1 [81,82,83]. Similar to inbred strains, SS pathology in these mice is characterized on the basis of infiltrates in SG and LG with the demonstration of SG functional loss. There are several other transgenic mice studied for SS pathology that are reviewed extensively by others [22].

In MRL/lpr mice LG pathology is described extensively. These mice have a mutation in fas^lpr^ (MRL/MpJ-*fas^lpr^/fas^lpr^*) that produces a time-dependent, spontaneously developing defective apoptosis of lymphocytes in peripheral lymphoid organs and systemic autoimmune disease [84,85]. In addition to SG pathology, anti-nuclear antibodies, SS specific anti-Ro, and La antibodies, MRL/lpr mice are characterized by LG pathology with predominant CD4+ lymphocytic infiltrates that express Th2 cytokines [86]. The observations from SG pathology in SS patients suggest that Th2 cytokines dominate the early phase as opposed to Th1 cytokines that are associated with the later phase of the disease [87,88]. Importantly, no corresponding evidence is available from LG pathology in SS patients. The LG infiltrates in MRL/lpr mice are predominantly detected in female mice, consistent with the predominance of SS in women [89]. Furthermore, analysis of the influence of sex on LG gene expression profile in MRL/lpr mice identified increased expression of genes related to inflammatory responses, antigen processing, and chemokines [90]. These genes represent an LG microenvironment that potentially drives the specific immune response observed in female mice. While LG infiltrates in MRL/lpr mice increase progressively with age, the overall nerve density in LG remains unaltered [48]. However, parasympathetic neural regulation of secretory response is disrupted by the LG inflammation via inhibition of the release of the neurotransmitter acetylcholine (Ach) [91]. This disruption is limited to nerves as LG secretory response to exogenously provided adrenergic and cholinergic stimulation is not affected [48]. The blockade of neural stimulation in LGs of MRL/lpr mice is attributed to the action of the pro-inflammatory cytokine IL-1β [92]. Additionally, LG inflammation in MRL/lpr mice also alters the contractile response of MEC to hormone oxytocin, known to induce contraction of MECs in mammary glands [93]. Thus similar to TSP-1−/− mice, results from MRL/lpr mice point to dysfunctional nerves and MECs as major contributors to the impairment of LG function. Overall, MRL/lpr, as well as most SS mouse models that focus on glandular pathology, do not evaluate the impact of the disease on ocular surface tissues or lacrimal functional unit as seen in SS patients.

## 3. Conclusions

While SG biopsies are included in all classification criteria developed for diagnosing SS, LG biopsies are included only in the Japanese classification criteria for SS [94]. A recent review of SS related literature in the context of ocular disease identified only 12 published studies since 1970 that describe histopathology of the LG in SS patients [3]. These limitations may explain the major focus on SG pathology and function in most SS mouse models. In the 100-year history of SS the detection of ocular surface damage appears as the earliest milestone [95], and both ocular symptoms and clinical signs are critical components of the diagnostic criteria for SS worldwide [96]. Considering that LG infiltration develops earlier in SS patients relative to SG infiltrates [97], it is possible that the ocular surface disease represents the earlier phase of SS. Therefore, the availability of animal models that include the ocular spectrum of SS will be highly useful in developing early interventions in SS, as well as closing the current gap in our understanding of how LG pathology in SS impacts ocular surface tissues. In the absence of an objective “gold standard” for defining ocular surface disease associated with SS, a recent study identified ocular staining score to be the diagnostic test with a high specificity and sensitivity to differentiate SS related ocular surface disease [18]. This ocular staining score reflects the disruption of corneal and conjunctival epithelial barriers. As such, the inclusion of equivalent ocular surface assessment in mouse models of SS in combination with LG pathology can represent the impairment to lacrimal functional unit more accurately while providing critical insights into the disease progression. As summarized in this review, very few mouse models of SS describe ocular surface disease. Investigations in these models would not only help reveal potential mechanisms underlying the ocular sicca symptoms in SS patients, but also help advance clinical translation by identifying novel targets in the treatment of SS. For example, analysis of LG pathology in TSP-1−/− and MRL/lpr mice suggests targeting neural regulation and MECs as potential approaches for future therapeutics.

Another aspect of clinical translation studies is the screening of new therapeutics for potential efficacy. Such proof-of-principle studies are critical in the early stages of drug development. Careful selection of a mouse model that recapitulates the SS pathology being targeted can strengthen the probability of success in clinical trials. For instance, the onset of the ocular surface disease in young animals and higher severity of the disease in NOD.Aire KO mice may be suitable to evaluate efficacies of drug candidates in later stages of the disease. Furthermore, validation of a model is crucial for clinical translation and demonstrating similarities in response to currently used treatments in SS patients represents a rigorous validation. Availability of such information in TSP-1−/− mice confirms their suitability as a preclinical model to represent SS related ocular pathology. Moreover, in this model, disease progression is monitored using objective tests that are also applicable in human subjects. This information may be relevant and advantageous in selecting appropriate endpoint measurements in clinical trials. In conclusion, the development of SS mouse models that closely recapitulate human ocular clinical manifestations is critical to the success of future drug development for the disease.

## Figures and Tables

**Table 1:** Objective tests used in SS diagnosis and observations related ocular surface disease in patients and animal models.

	SS Patients	TSP-1−/−	NOD strains			IL-2Rα (CD25) KO
			*Aec1Aec2*	H2^b^	Aire KO	
**Ocular Signs:**						
**Reduced tear volume**	✔	No change	Increased	✔	✔	✔
**Loss of corneal barrier integrity** (Increased corneal fluorescein score)	✔	✔	No change	Increased OGD staining	Filamentary keratopathy	Increased OGD staining
**Conjunctival damage** (Increased Lissamine green staining score) **/inflammation**	✔	✔	✔	✔	✔	✔
**Reduced salivary flow**	✔	NR	✔	✔	NR	✔
**SG histology** (Mononuclear infiltrates)	✔	✔ ^1^	✔	✔	✔	✔
**Autoantibodies** (Anti-SSA and Anti-SSB)	✔	✔	NR	NR	NR	NR
**Additional Observations**						
**LG histology** (Mononuclear infiltrates)	✔	✔	✔	✔	✔	✔
**Reduced goblet cell density**	✔	✔	✔	✔	NR	NR
**Reduced tear mucins**	✔	✔	NR	NR	NR	NR
**Therapeutic validation**	-	✔	NR	NR	NR	NR

^1^ Unpublished results; OGD, Oregon-Green Dextran488 conjugated dye; NR, not reported/tested. ✔ this is a check mark that means detection of the indicated ocular sign.—this is meant to indicate “not applicable”.

## Abbreviations

SS	Sjögren’s syndrome
LG	lacrimal gland
MEC	myoepithelial cells
NOD	non-obese diabetic
TSP-1	thrombospondin-1
